# Metacognitive disengagement and knowledge innovation quality in human–AI collaboration: the mediating role of cognitive offloading and the moderating roles of task complexity and AI literacy

**DOI:** 10.3389/fpsyg.2026.1975833

**Published:** 2026-10-28

**Authors:** Wei Xiao, Cheng-Hao Men, Jia-Cong Zhang

**Affiliations:** 1 College of Philosophy, Law & Political Science, Shanghai Normal University, Shanghai, China; 2 School of Management, Shandong University, Jinan, China

**Keywords:** AI literacy, cognitive offloading, human–AI collaboration, knowledge innovation quality, metacognitive disengagement, task complexity

## Abstract

Generative artificial intelligence is fundamentally reshaping the cognitive conditions of knowledge innovation. While human–AI collaboration improves the efficiency of knowledge production, it may also induce creators to outsourcing cognitive work to AI systems. This study conceptualized this tendency as task-level metacognitive disengagement, defined as the immediate reduction of planning, monitoring, and reflection within a specific AI-assisted task, and, drawing on metacognition theory, cognitive offloading theory and self-regulated learning theory, examined its impact on knowledge innovation quality, its psychological mechanism, and boundary conditions through four complementary experiments. Experiment 1 showed that higher AI assistance significantly increased metacognitive disengagement. Experiment 2 revealed that cognitive offloading partially mediated the relationship between AI assistance and metacognitive disengagement. Experiment 3 confirmed the negative effect of cognitive offloading on knowledge innovation quality, specifically on novelty, usefulness, and developability, with metacognitive disengagement partially mediating this effect. Experiment 4 tested the proposed moderated mediation model and found that task complexity strengthened, whereas individual AI literacy weakened, the negative indirect effect of cognitive offloading on knowledge innovation quality through metacognitive disengagement. These findings reveal a hidden cognitive cost behind the efficiency gains of human–AI collaboration and provide empirical evidence and theoretical implications for cultivating innovative talent and designing human–AI collaborative mechanisms in the era of artificial intelligence.

## 1 Introduction

Generative artificial intelligence is increasingly embedded in knowledge innovation activities. Large language models such as ChatGPT and DeepSeek can not only perform routine tasks such as information retrieval, text generation, and code writing, but also participate in conceptual ideation, solution design, and evaluative reasoning that have traditionally been regarded as core human cognitive activities (Dwivedi et al., 2023). Human–AI collaboration is becoming a new norm in knowledge production (Dell’Acqua et al., 2026). As AI systems take on more cognitive labor, however, a hidden concern arises: whether knowledge workers’ own cognitive engagement, especially the monitoring and regulation of their own thinking processes, is quietly receding (Raisch and Fomina, 2025).

Cognitive offloading theory suggests that humans have a fundamental tendency to transfer cognitive tasks to external tools to conserve limited cognitive resources (Risko and Gilbert, 2016). From writing tools to calculators, and from search engines to navigation systems, technological advances have continuously expanded the boundary of cognitive offloading (Logg et al., 2019). Generative AI represents a qualitative shift in this trajectory (Baldwin and von Hippel, 2011): it no longer merely assists memory or computation but increasingly undertakes higher-order cognitive operations such as inference, synthesis, evaluation, and generative ideation. This means that the focus of cognitive offloading is shifting from offloading memory to offloading thinking, a transition whose potential costs deserve close attention.

Metacognition refers to individuals’ awareness and regulation of their own cognitive processes, including planning, monitoring, and reflecting on cognitive activities (Flavell, 1979). Metacognitive ability not only influences learning efficiency but is also a key condition for deep thinking and creative problem solving (Schraw and Moshman, 1995). In knowledge innovation, individuals need to continuously survey problem spaces, evaluate intermediate solutions, and adjust cognitive strategies, all of which depend heavily on metacognitive engagement. When AI can instantly generate fluent, complete, and seemingly plausible solutions, however, innovators may reduce their advance planning of problem-solving paths, weaken their real-time monitoring of intermediate results, or even abandon critical reflection on final outputs. Salomon and Perkins (1989) long ago warned that the more intelligent a technology becomes, the more users tend to transfer thinking responsibility to the tool rather than using it to expand their own intellectual capacity. Generative AI has made this warning newly relevant. Recent work further suggests that knowledge production in human-machine collaboration is shifting from individual cognition toward human-machine composite systems, and that one of the central costs of this shift is the gradual loss of innovators’ agency and reflective space over their own cognitive processes (Chen, 2025).

Existing studies have primarily focused on the empowering effects of generative AI on learning performance, task efficiency, or the ideation stage of creativity (Cress and Kimmerle, 2023; Jia et al., 2024), while systematic changes in metacognitive engagement during knowledge innovation remain underexamined. The concept of “metacognitive laziness” has been proposed in learning contexts (Fan et al., 2025), but discussions have mostly centered on students using AI to bypass cognitive effort. Whether and how similar processes unfold in the more complex and open-ended context of knowledge innovation remains unclear (Raisch and Fomina, 2025). Knowledge innovation involves not only idea generation but also evaluation, selection, elaboration, and systematization, all of which place higher demands on metacognitive participation and are therefore particularly vulnerable to erosion through cognitive offloading.

Against this background, the present study introduces the concept of task-level metacognitive disengagement to capture the tendency of individuals to reduce or abandon planning, monitoring, and reflection over their own cognitive processes during human–AI collaborative knowledge innovation. We define task-level metacognitive disengagement as the immediate reduction of planning, monitoring, and reflection within a specific AI-assisted task. This construct is distinct from cognitive offloading: whereas offloading is a behavioral act of delegating cognitive tasks to an external tool, metacognitive disengagement is a metacognitive state reflecting reduced self-regulation of one’s own cognitive processes.

To answer these questions, we conducted four experiments. The first three experiments examined the main effect of AI assistance on metacognitive disengagement, the mediating role of cognitive offloading, and the causal impact on innovation quality. Because such effects are unlikely to be uniform across all situations, a fourth experiment further examined two boundary conditions that may strengthen or weaken the observed effects. Drawing on metacognition theory, cognitive offloading theory, and self-regulated learning theory (Winne and Hadwin, 1998), this research advances a process-oriented account of how human–AI collaboration shapes metacognitive engagement and knowledge innovation quality, with implications for the design of AI-assisted knowledge work and the cultivation of innovative talent.

## 2 Theoretical framework and hypotheses

### 2.1 Metacognitive disengagement: conceptualization and theoretical grounding

Metacognition refers to individuals’ awareness and regulation of their own cognitive processes, encompassing metacognitive knowledge and metacognitive experiences (Flavell, 1979). Later work has operationalized metacognition as a set of observable cognitive activities, including planning, monitoring, and evaluation (Schraw and Moshman, 1995). In knowledge innovation, metacognition is particularly important: innovators must plan possible paths while exploring the problem space, monitor the validity of intermediate outcomes during solution generation, and reflect on the entire process after task completion. These metacognitive activities distinguish genuine innovation from simple information recombination or routine problem solving.

Knowledge creation theory similarly emphasizes that the continuous conversion between tacit and explicit knowledge depends on individuals’ active direction-setting and reflective participation (Nonaka, 1994). Metacognitive engagement is therefore central to the creative dialogue through which new knowledge emerges.

Cognitive offloading theory suggests that humans have a general tendency to transfer cognitive tasks to external tools to conserve limited cognitive resources (Risko and Gilbert, 2016). Offloading itself is a strategic behavior: after evaluating task demands and available resources, individuals deliberately delegate some cognitive operations to external systems in order to concentrate on more valuable or irreplaceable activities. From this perspective, cognitive offloading does not necessarily lead to cognitive decline and may even function as a form of cognitive enhancement.

The distinctive feature of generative AI, however, is that it shifts cognitive offloading from peripheral assistance to core substitution. This shift echoes the broader automation-augmentation paradox in AI research, in which the same technology can either replace or enhance human cognition depending on how it is used (Raisch and Krakowski, 2021). When AI can perform reasoning, synthesis, and solution generation that were previously core cognitive operations of the innovator, the individual faces not merely a strategic choice of whether to offload, but a motivational question of whether to continue thinking after offloading. If AI outputs are sufficiently fluent and superficially plausible, innovators may, over repeated use, gradually reduce their active engagement with their own cognitive processes. At this point, cognitive offloading can slide from a strategic behavior into a habitual dependency.

We introduce the concept of task-level metacognitive disengagement to capture the reduction of metacognitive activity that occurs within a specific AI-assisted task. Task-level metacognitive disengagement is defined as the immediate reduction of planning, monitoring, and reflection over one’s own cognitive processes during a particular knowledge innovation task. This construct comprises three interrelated but distinguishable dimensions: (1) planning reduction, referring to diminished advance planning of problem-solving paths, strategy selection, and resource allocation before a task begins; (2) monitoring weakening, referring to reduced real-time checking of intermediate results, cognitive biases, and deviations from direction during task execution; and (3) reflection absence, referring to reduced retrospective review and evaluation of output quality, process effectiveness, and strategy transferability after task completion. Together, these dimensions capture the withdrawal of metacognitive engagement from active regulation.

It is important to distinguish task-level metacognitive disengagement from cognitive offloading and metacognitive laziness. Cognitive offloading is a behavioral construct: it refers to the act of delegating cognitive tasks to an external tool to optimize cognitive resource allocation (Risko and Gilbert, 2016). Task-level metacognitive disengagement, in contrast, is a metacognitive construct: it refers to the reduction of self-regulatory engagement with one’s own cognitive processes within a specific task. An individual may delegate a task to AI (high offloading) while still monitoring their own understanding and evaluating AI outputs (low disengagement), or may engage in moderate offloading while abandoning metacognitive oversight (high disengagement). Recent work further distinguishes between dependent and autonomous forms of cognitive offloading to generative AI, suggesting that not all offloading has identical cognitive consequences (Zhu et al., 2026). The two constructs are therefore conceptually distinct. Metacognitive laziness, used mainly in learning contexts, describes learners’ tendency to use AI as a shortcut to avoid effortful self-regulation (Fan et al., 2025). Task-level metacognitive disengagement differs in that it does not require conscious avoidance; it can emerge gradually as learners become accustomed to AI-generated outputs.

Within the construct itself, task-level metacognitive disengagement refers to the immediate reduction of planning, monitoring, and reflection within a specific AI-assisted task, whereas habitual metacognitive disengagement would refer to a stable, cumulative decline in metacognitive regulation that develops through repeated AI reliance over time. The present experiments focus on the former. Importantly, task-level metacognitive disengagement does not mean that individuals stop thinking altogether, nor that AI outputs are accepted without question. It describes a systematic decline in the quantity and depth of metacognitive investment: individuals may still evaluate the local plausibility of AI outputs, but they are less likely to question underlying assumptions, compare alternative solutions, or reflect on their own cognitive strategies. This partial engagement is precisely what makes task-level metacognitive disengagement difficult to detect while still undermining the deep cognitive processing required for knowledge innovation (Salomon and Perkins, 1989; Amabile, 1983).

### 2.2 AI assistance and metacognitive disengagement

Human–AI collaboration reshapes the cognitive architecture of knowledge innovation. When AI systems take over information retrieval, framework generation, and content organization, the immediate cognitive burden on the individual decreases. Yet this relief from cognitive load may carry an unintended metacognitive cost. In knowledge innovation, where tasks are open-ended and solutions must be actively constructed rather than passively received, the reduction of cognitive effort may coincide with a reduction in the self-regulatory processes that guide and evaluate one’s own thinking.

From the perspective of cognitive load, AI assistance reduces external cognitive load by taking over tasks that would otherwise demand substantial mental effort. In knowledge innovation, however, the cognitive resources freed by AI assistance do not automatically flow into deeper metacognitive processing. Instead, individuals may redirect those resources away from the task, reducing their planning of problem-solving paths, monitoring of intermediate results, and reflection on the overall process. Salomon and Perkins (1989) observed that the more intelligent a technology appears, the more users tend to transfer thinking responsibility to the tool rather than using it to extend their own intellectual capacity. Generative AI amplifies this tendency because its outputs are not only available but also fluent, complete, and superficially reasonable. Innovators may therefore accept AI-generated directions with less scrutiny, thereby weakening the metacognitive engagement that knowledge innovation demands.

From the perspective of self-regulated learning, Winne and Hadwin (1998) proposed a four-phase model comprising task definition, goal setting and planning, strategy enactment, and metacognitive adaptation. Metacognitive activity runs through each phase. In knowledge innovation, these phases are particularly consequential because the problem space is ill-defined and solutions require iterative evaluation. When AI can generate solutions rapidly, however, learners and innovators may skip the first two phases and move directly to strategy enactment, or skip the final adaptation phase and forgo critical reflection on the output. As a result, higher AI assistance increases the likelihood that individuals bypass the cognitive stages that require metacognitive participation.

Accordingly, we propose:

*H1*: AI assistance in human–AI collaboration positively affects metacognitive disengagement.

### 2.3 The mediating role of cognitive offloading

How does AI assistance lead to metacognitive disengagement? We propose that cognitive offloading is the core psychological mechanism linking the two.

Risko and Gilbert (2016) showed that individuals tend to offload cognitive tasks to external tools when facing high cognitive load. In human–AI collaborative knowledge innovation, AI systems undertake core cognitive tasks such as information retrieval, framework construction, and content generation with high efficiency and fluency. This outsourcing of thinking reduces immediate cognitive load and improves task efficiency, but it also gradually removes individuals from direct participation in and control over their own cognitive processes.

More importantly, cognitive offloading exerts a dual erosion effect on metacognition. First, offloading reduces the raw material for metacognitive monitoring. Metacognitive monitoring depends on perceiving and evaluating intermediate states of one’s own cognitive process; when individuals no longer personally filter information, construct frameworks, or generate content, they lose the cognitive material needed to monitor these processes. Second, offloading weakens the perceived necessity of metacognitive monitoring. When AI is perceived as highly reliable, the motivation to check one’s own thinking decreases (Glikson and Woolley, 2020). Cognitive offloading thus reduces metacognitive engagement and constitutes the core mechanism through which metacognitive disengagement emerges.

Accordingly, we propose:

*H2*: Cognitive offloading mediates the relationship between AI assistance and metacognitive disengagement.

### 2.4 Metacognitive disengagement and knowledge innovation quality

Metacognitive disengagement should impair knowledge innovation quality because of the functions that metacognition serves in the innovation process.

Metacognition contributes to knowledge innovation through three interrelated functions. Planning allows innovators to survey the problem space, anticipate possible solution paths, and commit to a direction before investing effort (Flavell, 1979). Monitoring enables them to evaluate intermediate results, detect deviations, and adjust course during the innovation process (Flavell, 1979). Reflection, finally, allows them to review the entire process after task completion and extract transferable strategies and insights (Winne and Hadwin, 1998).

When metacognitive disengagement occurs, these functions are compromised: innovators may accept the first plausible AI-generated solution without exploring alternatives (von Hippel, 2005), persist along unproductive paths without correcting course, or fail to learn from the episode and improve iteratively. These breakdowns map directly onto the three dimensions of knowledge innovation quality. Insufficient planning reduces novelty, because the problem space is not fully explored and alternative solutions are not seriously considered. Weakened monitoring reduces usefulness, because intermediate results are not critically evaluated and errors go uncorrected. Absent reflection limits developability, because the innovator does not extract lessons that would support iterative improvement. Together, these effects produce a systematic decline in innovation quality (Amabile, 1983).

It is worth noting that this decline may be masked in the short term by the apparent efficiency of AI outputs. Individuals may mistake fast access to an answer for high-quality innovation—an illusion of competence that is particularly insidious because it prevents individuals from recognizing the erosion of their own cognitive capacity.

Accordingly, we propose:

*H3*: Metacognitive disengagement negatively affects knowledge innovation quality.

### 2.5 The moderating roles of task complexity and AI literacy

The negative effect of metacognitive disengagement on knowledge innovation quality is unlikely to be uniform across all conditions. We propose task complexity and individual AI literacy as two key boundary conditions.

We selected these moderators over other plausible candidates such as cognitive style, openness, AI trust, or task type for two reasons. First, task complexity directly determines the intensity of metacognitive processing required for innovation and is therefore a core task characteristic that explains variation in the consequences of metacognitive disengagement. Individual AI literacy directly affects how individuals process and depend on AI outputs and is closely linked to the formation and consequences of metacognitive disengagement. Second, both factors are practically modifiable: task complexity can be controlled through workflow design, and AI literacy can be improved through education and training.

Task complexity refers to the intensity and structural complexity of cognitive effort required by a knowledge innovation task (Campbell, 1988). When tasks are simple, AI can provide relatively complete solutions, and the necessity of metacognitive engagement is low; the negative effect of metacognitive disengagement is therefore limited. When tasks are highly complex, however, AI outputs typically serve only as a starting point. Individuals must critically evaluate, integrate across domains, and deeply process AI-generated content. Under such conditions, abandoning these deep-processing activities due to metacognitive disengagement should produce greater damage to innovation quality. Task complexity should thus strengthen the negative effect of metacognitive disengagement on innovation quality.

AI literacy refers to individuals’ knowledge, skills, and attitudes for understanding, using, and critically evaluating AI systems (Long and Magerko, 2020). Recent work has further developed validated scales for collaborative AI literacy and collaborative AI metacognition, highlighting the metacognitive dimension of effective human–AI collaboration (Sidra and Mason, 2025). Individuals with high AI literacy are more likely to treat AI as a cognitive augmentation tool rather than a substitute for thinking, to critically examine AI outputs, and to maintain metacognitive vigilance during human–AI collaboration. By contrast, individuals with low AI literacy are more likely to rely on AI uncritically. AI literacy should therefore buffer the negative effect of metacognitive disengagement on innovation quality.

We further specify that these moderating effects operate on the indirect path from cognitive offloading through metacognitive disengagement to knowledge innovation quality, rather than merely on the direct relation between metacognitive disengagement and innovation quality.

Accordingly, we propose:

*H4*: Task complexity positively moderates the indirect effect of cognitive offloading on knowledge innovation quality through metacognitive disengagement. That is, the negative indirect effect is stronger when task complexity is high.

*H5*: AI literacy negatively moderates the indirect effect of cognitive offloading on knowledge innovation quality through metacognitive disengagement. That is, the negative indirect effect is weaker when AI literacy is high.

### 2.6 Theoretical model

Figure 1 presents the proposed moderated mediation model. AI assistance is the independent variable; cognitive offloading and metacognitive disengagement are sequential mediators; knowledge innovation quality is the outcome variable; and task complexity and AI literacy are moderators. The core path is that AI assistance increases cognitive offloading, which in turn strengthens metacognitive disengagement and ultimately impairs knowledge innovation quality. The strength of this mediated path depends on task complexity and AI literacy.

**Figure 1:**
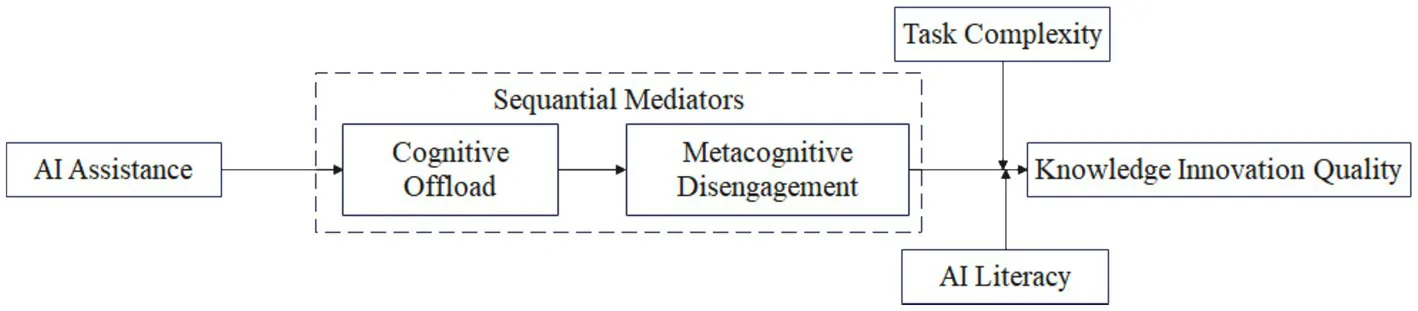
Theoretical model of this study. Paths tested as following: Experiment 1: AI assistance → metacognitive disengagement; Experiment 2: AI assistance → cognitive offloading → metacognitive disengagement; Experiment 3: cognitive offloading → metacognitive disengagement → knowledge innovation quality; Experiment 4: moderating roles of task complexity and AI literacy on the second-stage path.

The model was tested through four complementary experiments rather than within a single study. Experiment 1 examined the main effect of AI assistance on metacognitive disengagement. Experiment 2 tested the mediating role of cognitive offloading. Experiment 3 examined the effect of manipulated cognitive offloading on knowledge innovation quality through metacognitive disengagement. Experiment 4 tested the moderating roles of task complexity and AI literacy on the second-stage path. This multi-study strategy allowed each segment of the model to be examined with greater precision than a single omnibus design, although the complete sequential pathway was not directly estimated within one dataset.

## 3 Experiment 1: the main effect of AI assistance on metacognitive disengagement

Experiment 1 was designed to test the main effect of AI assistance on metacognitive disengagement in human–AI collaborative knowledge innovation. Using a single-factor two-level between-subjects design (AI assistance: high vs. low), we compared participants’ metacognitive disengagement in the same knowledge innovation task under the two assistance conditions.

### 3.1 Method

#### 3.1.1 Participants

Sample size was determined with G*Power 3.1 (Faul et al., 2007). With α = 0.05, a medium effect size *d* = 0.5, and power 1 – *β* = 0.80 for an independent-samples *t-*test, at least 51 participants per group were required. Accounting for potential invalid data in online experiments, participants were recruited through Credamo, an online data collection platform. Invalid responses were screened out in real time through attention checks, and data collection continued until 150 valid responses were obtained. Participants included 68 men (45.3%) and 82 women (54.7%), with a mean age of 28.64 years (SD = 6.72). They were randomly assigned to a high-assistance condition (*n* = 75) or a low-assistance condition (*n* = 75). All participants provided informed consent and received compensation after the experiment.

#### 3.1.2 Design and procedure

The experiment used a single-factor two-level between-subjects design. Participants were randomly assigned to either the high-assistance or the low-assistance condition and completed a smart home product innovation proposal task in a unified experimental platform.

An AI assistant was presented through a chat interface. The assistant’s responses were generated from researcher-configured prompt templates and a task-domain knowledge base to ensure that all participants within each condition received a consistent interactive experience. In the high-assistance condition, the AI assistant provided relatively complete solution-oriented responses to task-related requests, including generating target user profiles, offering reference directions for product features, and commenting on participants’ proposals. In the low-assistance condition, the AI assistant provided only brief responses to factual information queries and did not proactively offer creative suggestions or solution content. The task requirements were identical across conditions; only the degree of AI assistance differed.

A pilot study was conducted before the formal experiment to calibrate task clarity, the distinctiveness of the AI response strategies, and the reasonableness of the task completion time. Pilot data were not included in the formal analysis.

After reading the task materials, participants completed an attention check by briefly describing the kind of help the AI assistant would provide. Responses clearly inconsistent with the task instructions were excluded. Participants could ask the AI assistant questions during the task. After completing the task, they filled out the metacognitive disengagement scale and the manipulation check measure.

#### 3.1.3 Measures

##### 3.1.3.1 Task-level metacognitive disengagement

To assess task-level metacognitive disengagement, we developed a nine-item scale grounded in classic metacognition research that identifies planning, monitoring, and reflection as core metacognitive activities (Flavell, 1979; Schraw and Moshman, 1995; Winne and Hadwin, 1998). Its item format was informed by the measurement approach to metacognitive laziness in learning contexts (Fan et al., 2025) and adapted to the knowledge innovation context. The scale contains three dimensions, each measured with three items: planning reduction (e.g., “In this task, I rarely spent time planning the steps to solve the problem”), monitoring weakening (e.g., “In this task, I did not continuously check whether my thinking was correct”), and reflection absence (e.g., “After completing the task, I did not review and summarize my thinking process”), to capture the immediate reduction of these activities within the specific AI-assisted task rather than a general tendency. Items were rated on a 7-point scale (1 = strongly disagree, 7 = strongly agree). Higher scores indicated stronger metacognitive disengagement. In this experiment, Cronbach’s α was 0.89.

##### 3.1.3.2 AI assistance manipulation check

Perceived AI assistance was measured with 3 items: “The AI assistant provided me with substantial help,” “The AI assistant undertook a large part of my thinking work,” and “The AI assistant provided substantive suggestions.” Items were rated on a 7-point scale (1 = strongly disagree, 7 = strongly agree). Higher scores indicated greater perceived AI assistance. Cronbach’s α was 0.86.

Participants also reported gender, age, education level, and familiarity with AI. Two attention check items were randomly embedded in the questionnaire. To reduce demand characteristics, an open-ended question at the end asked participants what they thought the purpose of the experiment was.

#### 3.1.4 Scale validation

Because the scale of task-level metacognitive disengagement was newly developed, we examined its psychometric properties before using it in the main analyses.

First, content validity was assessed by five experts in human-computer interaction, scale development, and knowledge community management. Each item was rated for relevance to its intended dimension on a 4-point scale, and the content validity index (CVI) exceeded 0.80 for all retained items.

Second, because the scale was administered identically across all four experiments, we combined the data from all four samples (*N* = 728) to conduct exploratory factor analysis (EFA, see Appendix B) and confirmatory factor analysis (CFA). The hypothesized three-factor model (planning reduction, monitoring weakening, reflection absence) showed acceptable fit: χ^2^(24) = 98.42, CFI = 0.95, TLI = 0.94, RMSEA = 0.06 (90% CI [0.04, 0.08]), SRMR = 0.04. All factor loadings were significant (*p* < 0.001) and ranged from 0.67 to 0.84. A one-factor model [χ^2^(27) = 386.21, CFI = 0.73, RMSEA = 0.15] and a two-factor model [χ^2^(26) = 214.57, CFI = 0.84, RMSEA = 0.11] both showed significantly worse fit, supporting the three-factor structure.

Third, convergent validity was supported by average variance extracted (AVE) values of 0.62 (planning reduction), 0.59 (monitoring weakening), and 0.61 (reflection absence), all above the 0.50 threshold, and by composite reliability values of 0.88, 0.87, and 0.88.

Fourth, discriminant validity was assessed by comparing the scale with the cognitive offloading scale used in Experiments 2, 3, and 4. The HTMT ratio between the two constructs was 0.61, below the 0.85 threshold, and the Fornell-Larcker criterion was satisfied: the square root of AVE for each dimension (0.79, 0.77, 0.78) exceeded the correlations between that dimension and cognitive offloading (ranging from 0.42 to 0.51).

Fifth, subscale reliabilities and correlations were examined. Cronbach’s alpha was 0.87 for planning reduction, 0.86 for monitoring weakening, and 0.87 for reflection absence. The three subscales were moderately correlated (*r*s = 0.45 to 0.52, *p*s < 0.001), supporting the multidimensional structure.

Measurement invariance across the four experiments is reported in the Appendix C. Overall, these results indicate that the nine-item task-level metacognitive disengagement scale is a reliable and valid instrument for capturing immediate reductions in planning, monitoring, and reflection within AI-assisted tasks.

#### 3.1.5 Data screening and preliminary analysis

Responses to the open-ended purpose question were coded. If a participant’s answer was highly consistent with the aim of measuring metacognitive disengagement, sensitivity analyses were planned by excluding such participants. In this experiment, no participants needed to be excluded based on this criterion.

### 3.2 Results

#### 3.2.1 Manipulation check

An independent-samples *t-*test showed that perceived AI assistance was significantly higher in the high-assistance condition (*M* = 5.67, SD = 1.12) than in the low-assistance condition (*M* = 3.85, SD = 1.38), *t*(148) = 8.94, *p* < 0.001, Cohen’s *d* = 1.46. The manipulation of AI assistance was effective.

#### 3.2.2 AI assistance and metacognitive disengagement

As shown in Table 1, participants in the high-assistance condition scored significantly higher on overall metacognitive disengagement than those in the low-assistance condition, t(148) = 4.12, *p* < 0.001, Cohen’s *d* = 0.67. The same pattern held for all three dimensions: planning reduction, *t*(148) = 3.96, *p* < 0.001, Cohen’s *d* = 0.64; monitoring weakening, *t*(148) = 3.94, *p* < 0.001, Cohen’s *d* = 0.64; and reflection absence, *t*(148) = 3.78, *p* < 0.001, Cohen’s *d* = 0.61. These results support H1.

**Table 1:** Comparison of metacognitive disengagement scores under different AI assistance levels (Experiment 1).

Variable	High assistance	Low assistance	*t*(148)	*p*	Cohen’s *d*
Overall metacognitive disengagement	4.82 (1.21)	3.96 (1.35)	4.12	<0.001	0.67
Planning reduction	4.91 (1.35)	4.02 (1.42)	3.96	<0.001	0.64
Monitoring weakening	4.76 (1.28)	3.89 (1.41)	3.94	<0.001	0.64
Reflection absence	4.80 (1.33)	3.97 (1.38)	3.78	<0.001	0.61

Standard deviations are in parentheses.

A one-way analysis of covariance (ANCOVA) was conducted with AI assistance as the independent variable, metacognitive disengagement as the dependent variable, and gender, age, education, and AI familiarity as covariates. After controlling for these covariates, the main effect of AI assistance remained significant, *F*(1, 144) = 16.85, *p* < 0.001, η^2^ = 0.105. Among the covariates, AI familiarity had a significant negative effect on metacognitive disengagement, *F*(1, 144) = 4.32, *p* = 0.039, η^2^ = 0.029, indicating that participants more familiar with AI showed lower metacognitive disengagement. The effects of gender, age, and education were not significant (*p*s > 0.10).

### 3.3 Discussion

Experiment 1 provided initial evidence for the main effect of AI assistance on metacognitive disengagement in human–AI collaborative knowledge innovation. Participants in the high-assistance condition showed significantly higher levels of planning reduction, monitoring weakening, and reflection absence than those in the low-assistance condition, supporting H1.

A particularly interesting finding was that greater familiarity with AI was associated with lower, rather than higher, metacognitive disengagement. This pattern runs counter to the intuitive expectation that familiarity breeds dependency. One possible explanation is that individuals who are more familiar with AI are also more aware of its limitations and therefore more likely to maintain critical engagement and metacognitive vigilance when using it. This interpretation points to AI literacy as a potentially important buffer against metacognitive disengagement, which informed the inclusion of individual AI literacy as a moderator in later experiments.

## 4 Experiment 2: the mediating role of cognitive offloading

Experiment 1 established the main effect of AI assistance on metacognitive disengagement but did not identify the underlying psychological mechanism. Experiment 2 extended the investigation in two ways. First, it changed the knowledge innovation task from product design to a service innovation proposal, thereby broadening the task context. Second, it introduced cognitive offloading as a measured mediator to test whether AI assistance increases metacognitive disengagement through greater cognitive offloading.

### 4.1 Method

#### 4.1.1 Participants

The sample size was determined using the same standards as in Experiment 1: *α* = 0.05, *d* = 0.5, and power 1 – *β* = 0.80, requiring at least 51 participants per group. Because mediation analyses typically require larger samples than simple group comparisons, the sample size was increased. Participants were recruited through Credamo, with invalid responses screened out in real time through attention checks. The final sample comprised 162 valid participants: 73 men (45.1%) and 89 women (54.9%), with a mean age of 29.17 years (SD = 7.34). Participants were randomly assigned to a high-assistance condition (*n* = 82) or a low-assistance condition (*n* = 80).

#### 4.1.2 Design and procedure

The experiment used the same single-factor two-level between-subjects design as Experiment 1. In the community service innovation proposal task, participants were asked to design an innovative service solution addressing the daily needs of elderly and child populations in urban communities. This task was selected to extend the product innovation context of Experiment 1 to a service innovation context, thereby increasing the generalizability of the findings. The task requirements, AI assistant response strategies, attention checks, and experimental procedure were identical to those in Experiment 1.

After completing the task, participants filled out the cognitive offloading scale, the metacognitive disengagement scale, and the AI assistance manipulation check, followed by the same open-ended question about the perceived purpose of the experiment.

#### 4.1.3 Measures

##### 4.1.3.1 Cognitive offloading

The cognitive offloading scale was adapted from the cognitive offloading framework of Risko and Gilbert (2016) and contextualized for human–AI collaboration. It comprised 4 items: “In this task, I delegated a large part of my thinking to the AI,” “In this task, I let the AI complete content that required mental effort,” “In this task, I reduced my own effort in processing complex information,” and “In this task, I used the AI to reduce my cognitive load.” Items were rated on a 7-point scale (1 = strongly disagree, 7 = strongly agree), with higher scores indicating greater cognitive offloading. Cronbach’s α was 0.87.

##### 4.1.3.2 Task-level metacognitive disengagement

The task-level metacognitive disengagement scale was identical to that used in Experiment 1. Cronbach’s α was 0.88.

##### 4.1.3.3 AI assistance manipulation check

Perceived AI assistance was measured with the same 3 items as in Experiment 1. Cronbach’s α was 0.85.

#### 4.1.4 Data screening and preliminary analysis

Responses to the open-ended purpose question were coded using the same procedure as in Experiment 1. If a participant explicitly mentioned metacognitive engagement, thinking involvement, or AI dependence in their answer, they were flagged as a high-suspicion participant. The main analyses were then rerun after excluding these participants to examine whether the results remained stable. No high-suspicion participants were identified, and all participants included in the final analyses passed the attention checks.

Given that all measures in this experiment were self-reported, we also conducted a Harman’s single-factor test to assess common method bias. The first unrotated factor accounted for 31.4% of the variance, which is below the 50% threshold, adding correlations among measures were moderate (*r*s = 0.18–0.52), suggesting that common method bias was not a serious concern.

### 4.2 Results

#### 4.2.1 Manipulation check

Perceived AI assistance was significantly higher in the high-assistance condition (*M* = 5.73, SD = 1.08) than in the low-assistance condition (*M* = 3.92, SD = 1.35), *t*(160) = 9.48, *p* < 0.001, Cohen’s *d* = 1.49. The manipulation was effective.

#### 4.2.2 AI assistance and metacognitive disengagement

Consistent with Experiment 1, participants in the high-assistance condition scored significantly higher on metacognitive disengagement (*M* = 4.91, SD = 1.18) than those in the low-assistance condition (*M* = 4.03, SD = 1.32), *t*(160) = 4.50, *p* < 0.001, Cohen’s *d* = 0.71.

#### 4.2.3 AI assistance and cognitive offloading

Participants in the high-assistance condition also scored significantly higher on cognitive offloading (*M* = 5.12, SD = 1.05) than those in the low-assistance condition (*M* = 3.87, SD = 1.29), *t*(160) = 6.83, *p* < 0.001, Cohen’s *d* = 1.08. Thus, higher AI assistance was associated with stronger cognitive offloading.

#### 4.2.4 Mediation analysis

Mediation was tested using the PROCESS macro (Model 4; Hayes, 2013), with AI assistance as the independent variable (high = 1, low = 0), cognitive offloading as the mediator, and metacognitive disengagement as the dependent variable. Bootstrap resampling with 5,000 iterations was used to estimate bias-corrected 95% confidence intervals. Results are summarized in Table 2.

**Table 2:** Mediating effect of cognitive offloading: Bootstrap analysis (Experiment 2).

Path	*B*	SE	95%CI	*p*
Total effect(c)	0.88	0.20	[0.49, 1.27]	<0.001
Direct effect(c’)	0.37	0.15	[0.08, 0.66]	0.012
Indirect effect(a × b)	0.51	0.10	[0.32, 0.72]	—

Bootstrap resamples = 5,000; a confidence interval excluding zero indicates a significant effect. “—” indicates that effect does not have a *p-*value reported.

The total effect of AI assistance on metacognitive disengagement was significant, *c* = 0.88, SE = 0.20, *p* < 0.001, 95% CI [0.49, 1.27]. AI assistance significantly predicted cognitive offloading, *a* = 1.25, *p* < 0.001, and cognitive offloading significantly predicted metacognitive disengagement, *b* = 0.41, *p* < 0.001. The indirect effect was significant, a × b = 0.51, SE = 0.10, 95% CI [0.32, 0.72], excluding zero. After controlling for the mediator, the direct effect of AI assistance on metacognitive disengagement remained significant, c’ = 0.37, SE = 0.15, *p* = 0.012, 95% CI [0.08, 0.66]. The indirect effect accounted for 58.0% of the total effect, indicating partial mediation.

As a robustness check, a stepwise regression analysis was conducted following the procedures recommended by Wen et al. (2004). AI assistance significantly predicted cognitive offloading (*β* = 0.48, *p* < 0.001), cognitive offloading significantly predicted metacognitive disengagement (*β* = 0.39, *p* < 0.001), and the direct effect of AI assistance on metacognitive disengagement decreased from *β* = 0.42 (*p* < 0.001) to *β* = 0.18 (*p* = 0.012) after including cognitive offloading. These results were consistent with the bootstrap analysis. Figure 2 displays the mediation paths.

**Figure 2:**
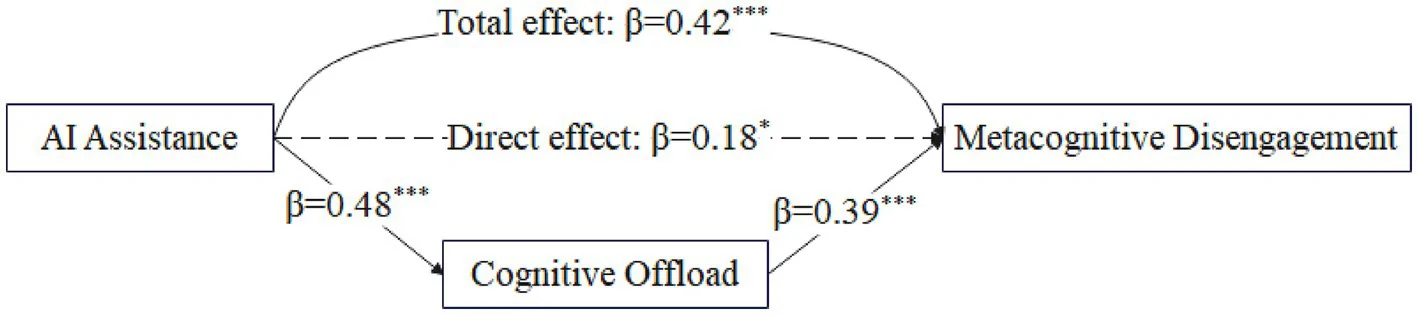
Mediating role of cognitive offloading: Stepwise regression analysis.

### 4.3 Discussion

Experiment 2 replicated the main effect of AI assistance on metacognitive disengagement in a different task context and provided evidence for the mediating role of cognitive offloading. Higher AI assistance increased cognitive offloading, which in turn strengthened metacognitive disengagement. Under high AI assistance, individuals outsourced core cognitive tasks such as information retrieval, framework construction, and solution generation, reducing their direct participation in the cognitive process. This offloading deprived metacognitive monitoring of its raw material and weakened the perceived necessity of self-checking, thereby producing a chain from cognitive offloading through reduced metacognitive engagement to metacognitive disengagement.

The finding that cognitive offloading only partially mediated the relationship suggests that other mechanisms may also be at work, such as changes in self-efficacy or perceived task responsibility. These possibilities remain open for future investigation.

## 5 Experiment 3: the causal effect of cognitive offloading on knowledge innovation quality

Experiment 2 provided evidence that cognitive offloading mediates the relationship between AI assistance and metacognitive disengagement. However, because cognitive offloading was measured rather than manipulated, the causal direction remained uncertain. Experiment 3 addressed this limitation by manipulating the degree of cognitive delegation to AI—the core behavioral expression of cognitive offloading in human–AI collaboration—and examining its causal effect on knowledge innovation quality, with metacognitive disengagement as a mediator.

### 5.1 Method

#### 5.1.1 Participants

The sample size was determined using the same criteria as in Experiment 1: *α* = 0.05, *d* = 0.5, and power 1 – *β* = 0.80, requiring at least 51 participants per group. Because expert ratings of innovation quality were involved and some data loss was possible, the sample size was increased. Participants were recruited through Credamo, and invalid responses were screened out in real time. The final sample comprised 176 valid participants: 80 men (45.5%) and 96 women (54.5%), with a mean age of 28.93 years (SD = 7.11). Participants were randomly assigned to a high-delegation condition (*n* = 88) or a low-delegation condition (*n* = 88).

#### 5.1.2 Design and procedure

The experiment used a single-factor two-level between-subjects design (workflow condition: high delegation vs. low delegation). Unlike Experiments 1 and 2, Experiment 3 did not manipulate AI assistance. Instead, cognitive offloading was manipulated through a high-delegation versus low-delegation workflow condition, implemented via task instructions and the response strategy of the AI assistant.

In an urban public space micro-renewal proposal task, participants were asked to design a micro-renewal plan for a public space in a city they were familiar with, including identifying core problems, proposing renewal strategies and design solutions, describing implementation steps and expected effects, and evaluating feasibility and innovativeness. This task was selected to extend the product and service innovation contexts of Experiments 1 and 2 to a spatial and social innovation context.

Participants in the high-delegation condition were instructed that they could make full use of the AI assistant to complete problem diagnosis, design direction reference, and solution refinement, with the goal of minimizing effortful thinking from scratch. Participants in the low-delagation condition were instructed to rely primarily on their own thinking for the core content of the solution and to use the AI assistant only for factual information queries. Both groups used the same AI assistant interface, but the response strategies differed: in the high-delagation condition, the AI assistant provided detailed responses to any task-related request; in the low-delagation condition, it provided only brief responses to factual queries about policies, statistics, or cases, and did not elaborate on creative requests. To reduce demand characteristics, the low-delagation instructions did not explicitly tell participants to complete the task independently. Instead, participants were asked to complete the core content through their own thinking, with the AI assistant providing only factual information support.

After reading the task materials, participants completed an attention check to confirm that they understood the scope of AI support. After completing the task, they filled out the cognitive offloading manipulation check and the metacognitive disengagement scale, and reported what they thought the purpose of the experiment was.

#### 5.1.3 Measures

##### 5.1.3.1 Cognitive offloading manipulation check

Three items were used: “In this task, I delegated my thinking work to the AI,” “In this task, I reduced my own thinking effort,” and “In this task, I let the AI complete the core content.” Items were rated on a 7-point scale (1 = strongly disagree, 7 = strongly agree). Cronbach’s α was 0.88.

##### 5.1.3.2 Task-level metacognitive disengagement

The task-level metacognitive disengagement scale was identical to that used in Experiment 1. Cronbach’s α in this experiment was 0.89.

##### 5.1.3.3 Knowledge innovation quality

Three experts with backgrounds in urban planning or community design independently rated the innovation quality of each proposal. The experts were blind to experimental conditions. Proposals were rated on 3 dimensions of novelty, usefulness, and developability using a 7-point scale. The mean score across the three dimensions served as the composite measure of knowledge innovation quality. The intraclass correlation coefficient (ICC) among the three raters was 0.84, indicating good interrater reliability.

#### 5.1.4 Data screening and preliminary analysis

Responses to the open-ended purpose question were coded. If a participant explicitly mentioned “checking whether I depend on AI” or “checking my thinking involvement,” the participant was flagged as a high-suspicion case. The main analyses were then rerun after excluding these participants to examine robustness. Sensitivity analyses produced results consistent with the main analyses based on the full sample, indicating that suspicion about the experimental purpose did not systematically bias the findings.

Given that cognitive offloading and metacognitive disengagement were measured with self-report scales in the same questionnaire, we conducted a Harman’s single-factor test. The first unrotated factor accounted for 33.2% of the variance, below the 50% threshold, suggesting that common method bias was not a major concern. Correlations among self-report measures were moderate (*r*s = 0.21–0.48), and the outcome variable was expert-rated (*r* = −0.39 with metacognitive disengagement), reducing the likelihood of common method inflation.

### 5.2 Results

#### 5.2.1 Manipulation check

An independent-samples *t-*test showed that the high-delagation group scored significantly higher on cognitive offloading (*M* = 5.34, SD = 1.02) than the low-delagation group (*M* = 3.76, SD = 1.21), *t*(174) = 9.42, *p* < 0.001, Cohen’s *d* = 1.43. The manipulation of cognitive offloading was effective.

#### 5.2.2 Cognitive offloading and metacognitive disengagement

Participants in the high-delagation condition scored significantly higher on metacognitive disengagement (*M* = 5.02, SD = 1.14) than those in the low-delagation condition (*M* = 3.87, SD = 1.28), *t*(174) = 6.34, *p* < 0.001, Cohen’s *d* = 0.95. Thus, higher cognitive offloading led to stronger metacognitive disengagement.

#### 5.2.3 Cognitive offloading and knowledge innovation quality

Participants in the high-delagation condition scored significantly lower on overall knowledge innovation quality (*M* = 3.95, SD = 1.08) than those in the low-delagation condition (*M* = 4.78, SD = 1.15), *t*(174) = 4.97, *p* < 0.001, Cohen’s *d* = 0.75. The same pattern emerged for all three dimensions: novelty, high-delagation *M* = 3.82 (SD = 1.21) versus low-delagation *M* = 4.69 (SD = 1.28), *t*(174) = 4.65, *p* < 0.001, Cohen’s *d* = 0.70; usefulness, high-delagation *M* = 4.01 (SD = 1.15) versus low-delagation *M* = 4.85 (SD = 1.22), *t*(174) = 4.73, *p* < 0.001, Cohen’s *d* = 0.71; and developability, high-delagation *M* = 4.02 (SD = 1.19) versus low-delagation *M* = 4.80 (SD = 1.24), *t*(174) = 4.28, *p* < 0.001, Cohen’s *d* = 0.64.

A one-way ANCOVA was conducted with cognitive offloading condition as the independent variable, knowledge innovation quality as the dependent variable, and gender, age, education, and AI familiarity as covariates. After controlling for these covariates, the main effect of cognitive offloading remained significant, *F*(1, 170) = 24.31, *p* < 0.001, η^2^ = 0.125. Among the covariates, AI familiarity had a significant positive effect on knowledge innovation quality, *F*(1, 170) = 4.87, *p* = 0.029, η^2^ = 0.028, suggesting that individuals more familiar with AI performed better on the innovation task. The effects of gender, age, and education were not significant (*p*s > 0.10).

#### 5.2.4 Mediation analysis

Mediation was tested with the PROCESS macro (Model 4; Hayes, 2013), using cognitive offloading condition as the independent variable (high = 1, low = 0), metacognitive disengagement as the mediator, and knowledge innovation quality as the dependent variable. Bootstrap resampling with 5,000 iterations was used to estimate bias-corrected 95% confidence intervals. Results are presented in Table 3.

**Table 3:** Mediating effect of metacognitive disengagement: Bootstrap analysis (Experiment 3).

Path	*B*	SE	95%CI	*p*
Total effect(c)	−0.83	0.17	[−1.16, −0.50]	<0.001
Direct effect(c’)	−0.35	0.14	[−0.63, −0.07]	0.015
Indirect effect(a × b)	−0.48	0.10	[−0.68, −0.30]	—

Bootstrap resamples = 5,000; a confidence interval excluding zero indicates a significant effect. “—” indicates that effect does not have a *p-*value reported.

The total effect of cognitive offloading on knowledge innovation quality was significant, c = −0.83, SE = 0.17, *p* < 0.001, 95% CI [−1.16, −0.50]. Cognitive offloading significantly predicted metacognitive disengagement, *a* = 1.15, *p* < 0.001, and metacognitive disengagement significantly predicted knowledge innovation quality, *b* = −0.42, *p* < 0.001. The indirect effect was significant, a × b = −0.48, SE = 0.10, 95% CI [−0.68, −0.30], excluding zero. After controlling for metacognitive disengagement, the direct effect of cognitive offloading on knowledge innovation quality remained significant, c’ = −0.35, SE = 0.14, *p* = 0.015, 95% CI [−0.63, −0.07]. The indirect effect accounted for 57.8% of the total effect, indicating partial mediation.

As a robustness check, stepwise regression analyses yielded results consistent with the bootstrap analysis: cognitive offloading significantly predicted metacognitive disengagement (*β* = 0.43, *p* < 0.001), metacognitive disengagement significantly predicted knowledge innovation quality (*β* = −0.39, *p* < 0.001), and the direct effect of cognitive offloading on knowledge innovation quality decreased from *β* = −0.42 (*p* < 0.001) to *β* = −0.18 (*p* = 0.015) after including the mediator. Figure 3 presents the mediation paths.

**Figure 3:**
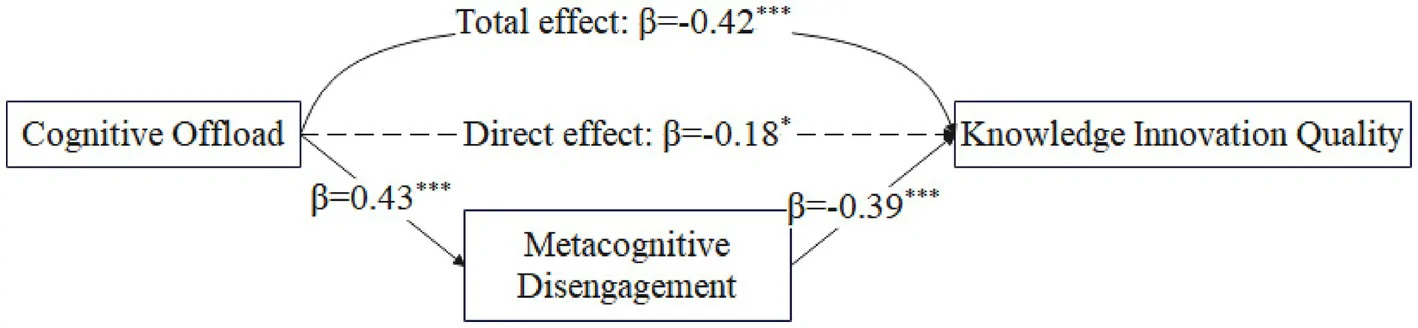
Mediating role of metacognitive disengagement: Stepwise regression analysis.

### 5.3 Discussion

Experiment 3 provided causal evidence for the effect of cognitive offloading on knowledge innovation quality through metacognitive disengagement. First, manipulating cognitive offloading produced the expected difference in metacognitive disengagement: participants in the high-delagation condition showed significantly stronger metacognitive disengagement than those in the low-delagation condition. Because cognitive offloading was experimentally manipulated rather than merely measured, these results strengthen the inference that workflow features involving greater delegation to AI lead to stronger metacognitive disengagement.

Second, cognitive offloading significantly impaired knowledge innovation quality across all three dimensions. Interestingly, the between-group difference was somewhat smaller for developability than for novelty and usefulness. One interpretation is that AI-generated solutions often appear structurally complete and operationally plausible in the short term, but lack the depth and sustainability that come from genuine iterative reflection. This pattern is consistent with the illusion of competence described by Fan et al. (2025), in which the fluent output of AI masks underlying deficiencies in quality (Tsakalerou et al., 2026).

Third, metacognitive disengagement partially mediated the effect of cognitive offloading on knowledge innovation quality. This finding indicates that cognitive offloading damages innovation quality primarily, though not exclusively, by weakening metacognitive planning, monitoring, and reflection. The residual direct effect also suggests that other mechanisms may operate alongside metacognitive disengagement. At the same time, the two workflow conditions inevitably differed in other features such as the detail of AI responses, the amount of independent effort requested, and the allocation of responsibility for core content. The findings should therefore be interpreted as reflecting the combined influence of a high-delegation workflow, within which cognitive offloading is the central feature. Future research could hold these features constant while varying only the degree of cognitive delegation to isolate the unique contribution of cognitive offloading. Consistent with Amabile’s (1983) componential theory of creativity, the results imply that when metacognitive skills are no longer exercised in human–AI collaborative tasks, the formal completeness of AI-generated output may conceal a loss of deeper creative quality.

## 6 Experiment 4: the moderating roles of task complexity and AI literacy

Experiments 1 through 3 established the main effect of AI assistance on metacognitive disengagement, the mediating role of cognitive offloading, and the causal impact of cognitive offloading on knowledge innovation quality through metacognitive disengagement. However, these effects may not be uniform across all conditions. Experiment 4 examined two boundary conditions of task complexity and individual AI literacy within the indirect effect from cognitive offloading through metacognitive disengagement to knowledge innovation quality. The experiment used a 2 (cognitive offloading: high vs. low) × 2 (task complexity: high vs. low) between-subjects design and also measured individual AI literacy as a continuous moderator.

### 6.1 Method

#### 6.1.1 Participants

Sample size was determined with G*Power 3.1 (Faul et al., 2007). For a two-way ANOVA with α = 0.05, medium effect size *f* = 0.25, and power 1 – *β* = 0.80, at least 128 participants were required. Because the study involved multiple experimental conditions, expert ratings, and moderated mediation analyses, the sample size was increased. Participants were recruited through Credamo, with invalid responses screened out in real time. The final sample comprised 240 valid participants: 108 men (45.0%) and 132 women (55.0%), with a mean age of 29.41 years (SD = 7.52). Participants were randomly assigned to one of four conditions, with 60 participants in each cell.

#### 6.1.2 Design and procedure

The experiment used a 2 (cognitive offloading: high vs. low) × 2 (task complexity: high vs. low) between-subjects design. The manipulation of cognitive offloading was identical to that in Experiment 3. In the high-delegation condition, the AI assistant provided detailed responses and participants were encouraged to rely on it extensively. In the low-delegation condition, participants were instructed to generate the core content themselves and to use the AI only for factual information.

Task complexity was manipulated by adjusting the structure and requirements of the innovation task. In the high-complexity condition, participants were asked to design an employee benefits plan for a medium-sized technology company with approximately 500 employees across multiple departments and age groups. They were required to consider diverse employee needs, departmental differences, budget constraints, long-term sustainability, and competitive differentiation, and to complete several components including need identification, proposal of three to five innovative measures, implementation planning with cost estimation, expected effects, and risk assessment. In the low-complexity condition, participants were asked to design a benefits plan for a small technology company with approximately 50 employees and were required only to propose three to five measures with a brief explanation of implementation. The two tasks shared the same theme but differed substantially in the diversity of target groups, number of constraints, and depth of analysis required. The manipulation strength was calibrated through pilot testing.

After reading the task materials, participants completed an attention check. After completing the task, they filled out the metacognitive disengagement scale, the task complexity manipulation check, the cognitive offloading manipulation check, and the AI literacy scale, and reported what they thought the purpose of the experiment was. Knowledge innovation quality was assessed through expert ratings.

#### 6.1.3 Measures

##### 6.1.3.1 Task-level metacognitive disengagement

The same 9-item task-level metacognitive disengagement scale from Experiment 1 was used. Cronbach’s α in this experiment was 0.89.

##### 6.1.3.2 Cognitive offloading manipulation check

The same 3-item measure from Experiment 3 was used. Cronbach’s α was 0.88.

##### 6.1.3.3 Task complexity manipulation check

Three items were used: “This task was very complex,” “Completing this task required a great deal of cognitive effort,” and “This task involved multiple factors that needed to be weighed.” Items were rated on a 7-point scale (1 = strongly disagree, 7 = strongly agree). Cronbach’s α was 0.85.

##### 6.1.3.4 AI literacy

AI literacy was measured with 6 items developed from the framework of Long and Magerko (2020), covering three dimensions: AI knowledge, AI use skills, and critical evaluation of AI. Example items include “I understand the basic working principles and boundaries of AI systems,” “I can effectively interact with AI systems to complete tasks,” and “I can identify potential biases and limitations in AI outputs.” Items were rated on a 7-point scale (1 = strongly disagree, 7 = strongly agree), with higher scores indicating greater AI literacy. Cronbach’s α was 0.88.

##### 6.1.3.5 Knowledge innovation quality

Three experts with backgrounds in human resource management or organizational behavior independently rated each proposal on novelty, usefulness, and developability using a 7-point scale. The experts were blind to experimental conditions. The mean of the 3 dimensions served as the composite measure of knowledge innovation quality. The intraclass correlation coefficient was 0.86, indicating good inter-rater reliability.

#### 6.1.4 Statistical strategy

Moderated mediation was tested with the PROCESS macro (Hayes, 2013). Because task complexity and AI literacy were theorized to moderate the relation between metacognitive disengagement and knowledge innovation quality (second-stage moderation), PROCESS Model 14 was used. Cognitive offloading condition was the independent variable (high = 1, low = 0), metacognitive disengagement was the mediator, and knowledge innovation quality was the dependent variable. Task complexity (high = 1, low = 0) and AI literacy (continuous, mean-centered) were entered separately as moderators. Bootstrap resampling with 5,000 iterations was used to estimate bias-corrected 95% confidence intervals. We also examined whether the interaction between cognitive offloading and each moderator predicted metacognitive disengagement to determine whether moderation occurred at the first stage.

#### 6.1.5 Data screening and preliminary analysis

Responses to the open-ended purpose question were coded. Participants whose answers explicitly mentioned metacognitive engagement, AI dependence, or innovation quality were flagged as high-suspicion cases. The main analyses were rerun after excluding these participants to examine robustness. Sensitivity analyses produced results consistent with the main analyses based on the full sample, indicating that suspicion about the experimental purpose did not systematically bias the findings. All participants included in the final analyses passed the attention checks.

Given that metacognitive disengagement and AI literacy were measured with self-report scales in the same questionnaire, we conducted a Harman’s single-factor test. The first unrotated factor accounted for 30.8% of the variance, below the 50% threshold. Correlations among self-report measures were moderate (*r*s = 0.16–0.45), and the outcome was expert-rated (*r* = −0.41). The moderate correlations and the use of expert ratings and experimental manipulation make common method bias an unlikely explanation for the findings.

### 6.2 Results

#### 6.2.1 Manipulation checks

The cognitive offloading manipulation was effective: the high-delagation group scored significantly higher on cognitive offloading (*M* = 5.41, SD = 1.04) than the low-delagation group (*M* = 3.69, SD = 1.18), *t*(238) = 12.04, *p* < 0.001, Cohen’s *d* = 1.55.

The task complexity manipulation was also effective: the high-complexity group perceived the task as significantly more complex (*M* = 5.56, SD = 1.10) than the low-complexity group (*M* = 3.82, SD = 1.34), *t*(238) = 11.03, *p* < 0.001, Cohen’s *d* = 1.42.

#### 6.2.2 Cognitive offloading and metacognitive disengagement: Main effect and first-stage moderation tests

A 2 × 2 ANOVA on metacognitive disengagement revealed a significant main effect of cognitive offloading, *F*(1, 236) = 39.82, *p* < 0.001, η^2^ = 0.144, with higher metacognitive disengagement in the high-delagation condition (*M* = 5.08, SD = 1.11) than in the low-delagation condition (*M* = 3.92, SD = 1.24). The main effect of task complexity was not significant, *F*(1, 236) = 1.24, *p* = 0.267, η^2^ = 0.005, nor was the interaction between cognitive offloading and task complexity, *F*(1, 236) = 0.85, *p* = 0.358, η^2^ = 0.004. Table 4 summarizes these results.

**Table 4:** ANOVA of cognitive offloading and task complexity on metacognitive disengagement (Experiment 4).

Source	*F*(1, 236)	*p*	η^2^
Cognitive offloading	39.82	<0.001	0.144
Task complexity	1.24	0.267	0.005
Cognitive offloading × Task complexity	0.85	0.358	0.004

To test whether AI literacy moderated the first-stage path, a regression was conducted with cognitive offloading, AI literacy, and their interaction as predictors of metacognitive disengagement. The interaction between cognitive offloading and AI literacy was not significant (*b* = −0.08, *p* = 0.421). Thus, neither task complexity nor AI literacy moderated the first-stage path from cognitive offloading to metacognitive disengagement, supporting the theorized second-stage moderation.

#### 6.2.3 The moderating role of task complexity

PROCESS Model 14 was used to test task complexity as a moderator of the indirect effect. Cognitive offloading significantly predicted metacognitive disengagement, *a* = 1.16, *p* < 0.001, and metacognitive disengagement significantly predicted knowledge innovation quality, *b* = −0.41, *p* < 0.001. The interaction between metacognitive disengagement and task complexity was significant, *b* = −0.34, *p* = 0.013, indicating that task complexity moderated the second-stage relation.

The index of moderated mediation was −0.39 (a × b = 1.16 × −0.34), 95% CI [−0.62, −0.16]. Conditional indirect effects showed that under low task complexity, the indirect effect of cognitive offloading on knowledge innovation quality through metacognitive disengagement was −0.48, 95% CI [−0.68, −0.28]; under high task complexity, the indirect effect was −0.87, 95% CI [−1.16, −0.58]. The index of moderated mediation was −0.39, 95% CI [−0.62, −0.16], excluding zero. Thus, task complexity strengthened the negative indirect effect, supporting H4. Table 5 presents these results.

**Table 5:** Moderated mediation analysis with task complexity as moderator (Experiment 4).

Path/Effect	B	*p*	95% CI
Cognitive offloading → Metacognitive disengagement (a)	1.16	<0.001	—
Metacognitive disengagement → Knowledge innovation quality (b)	−0.41	<0.001	—
Metacognitive disengagement × Task complexity	−0.34	0.013	—
Indirect effect at low task complexity	−0.48	—	[−0.68, −0.28]
Indirect effect at high task complexity	−0.87	—	[−1.16, −0.58]
Index of moderated mediation	−0.39	—	[−0.62, −0.16]

“—” indicates that *p-*values are not reported for bootstrap conditional indirect effects and the index of moderated mediation; significance is judged by whether the confidence interval excludes zero.

To further clarify the nature of this moderation, simple slope analyses (see Figure 4) were conducted. Under low task complexity, metacognitive disengagement significantly predicted lower knowledge innovation quality, *b* = −0.41, *p* < 0.001. Under high task complexity, the negative relation was significantly stronger, *b* = −0.75, *p* < 0.001. This pattern indicates that the detrimental effect of metacognitive disengagement on knowledge innovation quality was more pronounced when tasks were complex, consistent with H4.

**Figure 4:**
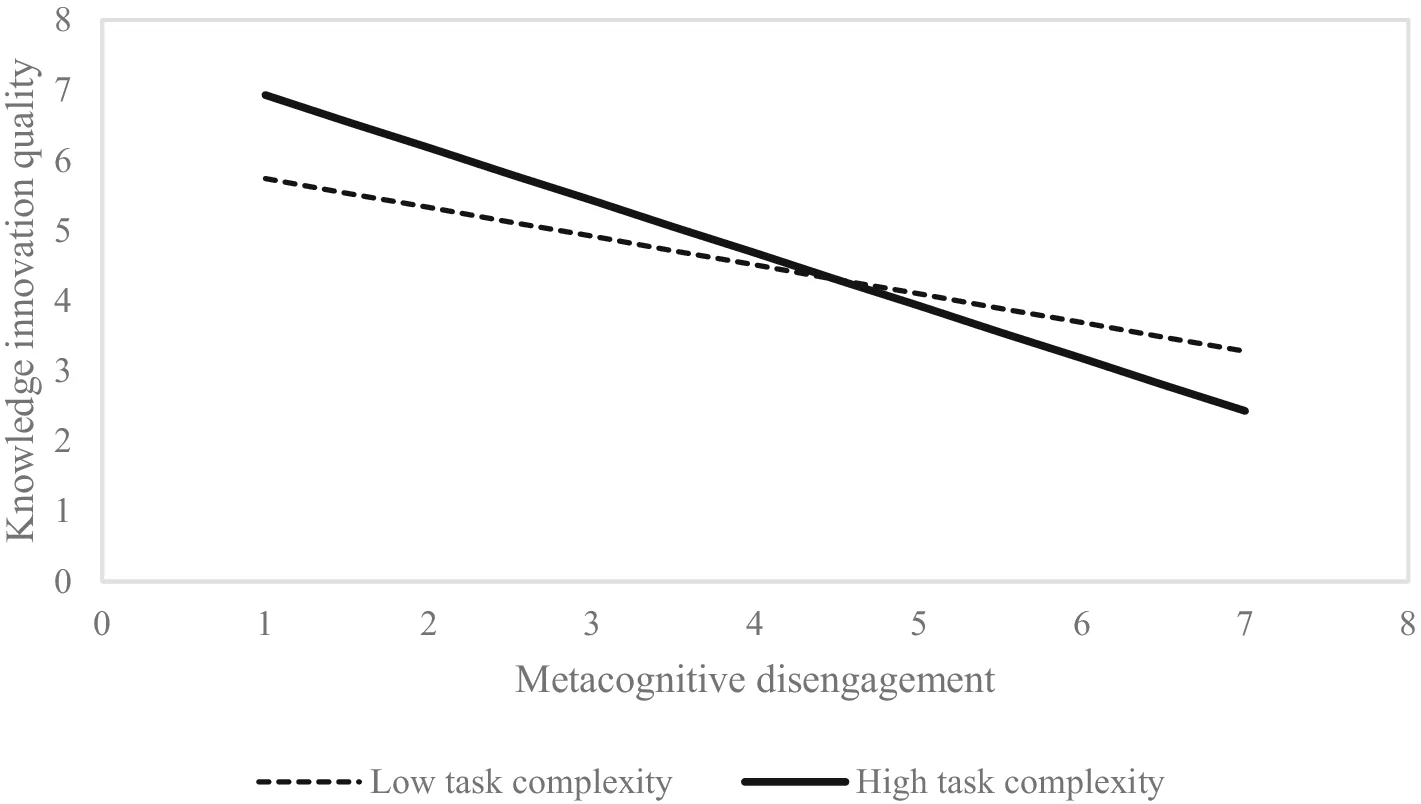
Moderating effect of task complexity on the relationship between metacognitive disengagement and knowledge innovation quality: simple slope analyses.

#### 6.2.4 The moderating role of AI literacy

A separate PROCESS Model 14 analysis was conducted with AI literacy as the moderator. Cognitive offloading significantly predicted metacognitive disengagement, *a* = 1.15, *p* < 0.001, and metacognitive disengagement significantly predicted knowledge innovation quality, *b* = −0.38, *p* < 0.001. The interaction between metacognitive disengagement and AI literacy was significant, *b* = 0.26, *p* = 0.006, indicating that AI literacy moderated the second-stage relation.

The index of moderated mediation was 0.30 (a × b = 1.15 × 0.26), 95% CI [0.09, 0.52]. Conditional indirect effects showed that at low AI literacy (mean minus one standard deviation), the indirect effect was −0.74, 95% CI [−1.02, −0.48]; at high AI literacy (mean plus one standard deviation), the indirect effect was −0.14, 95% CI [−0.30, 0.02]. The index of moderated mediation was 0.30, 95% CI [0.09, 0.52], excluding zero. Thus, AI literacy attenuated the negative indirect effect, supporting H5. Table 6 presents these results.

**Table 6:** Moderated mediation analysis with AI literacy as moderator (Experiment 4).

Path/Effect	B	*p*	95% CI
Cognitive offloading → metacognitive disengagement (a)	1.15	<0.001	—
metacognitive disengagement → Knowledge innovation quality (b)	−0.38	<0.001	—
metacognitive disengagement × AI literacy	0.26	0.006	—
Indirect effect at low AI literacy (−1 SD)	−0.74	—	[−1.02, −0.48]
Indirect effect at high AI literacy (+1 SD)	−0.14	—	[−0.30, 0.02]
Index of moderated mediation	0.30	—	[0.09, 0.52]

“—” indicates that *p-*values are not reported for bootstrap conditional indirect effects and the index of moderated mediation; significance is judged by whether the confidence interval excludes zero. AI literacy values refer to mean-centered scores.

To further illustrate the moderating role of AI literacy, a Johnson-Neyman analysis was conducted. The negative effect of metacognitive disengagement on knowledge innovation quality was significant when AI literacy was below 2.87 and became nonsignificant when AI literacy was above 4.63. Between 2.87 and 4.63, the confidence interval included zero, indicating that the conditional effect was not statistically significant, although the point estimate remained negative and gradually weakened. Figure 5 displays the conditional effect of metacognitive disengagement on knowledge innovation quality across levels of AI literacy.

**Figure 5:**
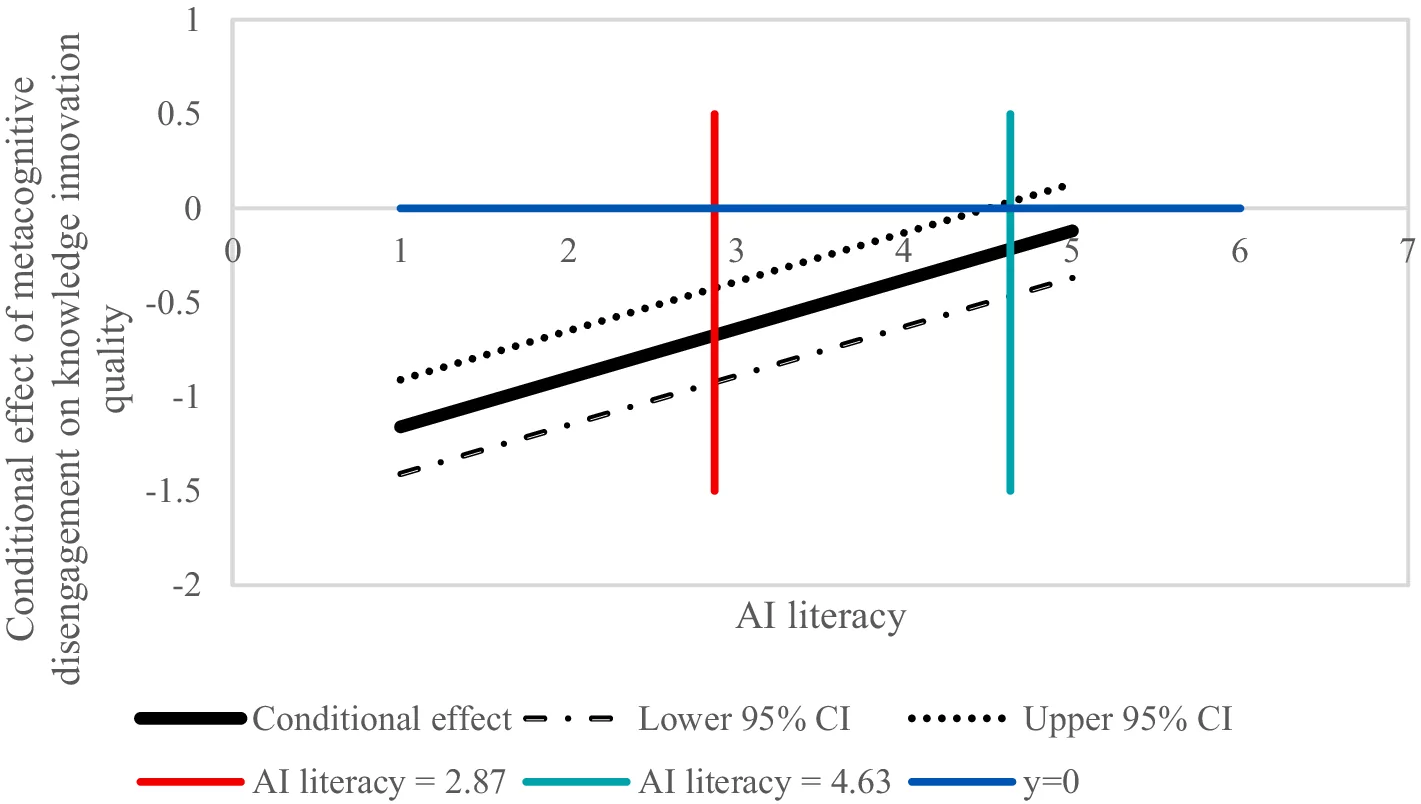
Moderating effect of AI literacy on the relationship between metacognitive disengagement and knowledge innovation quality: Johnson-Neyman plot.

### 6.3 Discussion

Experiment 4 extended the findings of Experiments 1 through 3 by identifying two boundary conditions of the indirect effect from cognitive offloading through metacognitive disengagement to knowledge innovation quality.

First, task complexity strengthened the negative indirect effect. Under high task complexity, the indirect effect of cognitive offloading on knowledge innovation quality through metacognitive disengagement was significantly stronger than under low task complexity. This pattern is consistent with the proposition that complex innovation tasks require more extensive metacognitive processing (Campbell, 1988). When tasks are complex, AI outputs typically serve only as a starting point, and individuals must critically evaluate, integrate, and deeply process AI-generated content. Under such conditions, metacognitive disengagement leads to particularly severe losses in innovation quality.

Second, AI literacy buffered the negative indirect effect. Individuals with higher AI literacy maintained relatively higher knowledge innovation quality even under high cognitive offloading, whereas those with lower AI literacy showed stronger negative effects. This finding suggests that the value of AI literacy lies not simply in better technical operation of AI but in the ability to maintain critical distance from AI outputs and to preserve metacognitive vigilance during human–AI collaboration (Long and Magerko, 2020).

Third, the supplementary analyses clarified that neither task complexity nor AI literacy moderated the first-stage path from cognitive offloading to metacognitive disengagement. Instead, both moderators operated at the stage at which metacognitive disengagement translated into innovation quality. This distinction indicates that metacognitive disengagement may arise fairly generally under AI-assisted conditions, but its consequences depend on task characteristics and individual capabilities. For individuals with high AI literacy or for simple tasks, even if metacognitive disengagement occurs, its actual damage to innovation quality is relatively limited; for complex tasks or low-AI-literacy individuals, the same level of metacognitive disengagement leads to more substantial quality loss.

At the same time, as in Experiment 3, the manipulation of cognitive offloading was implemented through a compound workflow condition. The high- and low-delegation conditions differed not only in the degree of cognitive delegation but also in the detail of AI responses, the amount of independent effort requested, and the allocation of responsibility for core content. The findings should therefore be interpreted as reflecting the combined influence of a high-delegation workflow, within which cognitive offloading is the central feature. Future research could hold these features constant while varying only the degree of cognitive delegation to isolate the unique contribution of cognitive offloading.

## 7 General discussion

The present research examined whether and how human–AI collaboration in knowledge innovation gives rise to metacognitive disengagement, a subtle cognitive cost that has received little attention in prior work. Across four experiments, we found consistent evidence that higher AI assistance increases metacognitive disengagement (Experiment 1), that this effect is partially mediated by cognitive offloading (Experiment 2), that manipulated cognitive offloading causally impairs knowledge innovation quality through metacognitive disengagement (Experiment 3), and that this mediated pathway is stronger when tasks are more complex but weaker among individuals with higher AI literacy (Experiment 4). Together, the findings support a moderated mediation model in which the efficiency gains of AI-assisted knowledge work are accompanied by a measurable decline in the metacognitive engagement that underpins high-quality innovation.

### 7.1 Key findings and theoretical interpretation

The central insight emerging from this research is that generative AI does not merely change how quickly or easily knowledge work is performed; it can also change the relation between creators and their own cognitive processes. When AI systems produce fluent, complete, and seemingly reasonable outputs, individuals reduce not only routine cognitive operations but also the planning, monitoring, and reflection that would otherwise accompany their own intellectual work. Importantly, metacognitive disengagement does not imply the absence of thought. Rather, it describes a systematic decline in the depth of metacognitive investment: individuals may still read, select, and even revise AI outputs, but they are less likely to question underlying assumptions, compare alternatives, or reflect on their own strategies. This partial engagement is precisely what makes metacognitive disengagement difficult to observe, yet sufficient to erode the deeper cognitive processing required for knowledge innovation.

These findings extend the classic argument of Salomon and Perkins (1989) that intelligent technologies can invite users to transfer thinking responsibility to the tool. Generative AI makes this tendency more consequential because it can perform not only peripheral cognitive tasks but also core operations such as inference, synthesis, and evaluation. The resulting shift from offloading memory to offloading thinking represents a qualitatively new phase of cognitive offloading (Risko and Gilbert, 2016). Our results suggest that the most important cost of this shift may not be a direct decline in task performance, but a gradual retreat from the metacognitive regulation that sustains long-term creative capability.

From a metacognition perspective, the present findings extend prior work that has linked metacognitive processes to complex problem solving and creative performance (Flavell, 1979; Schraw and Moshman, 1995). We show that in human–AI collaboration, metacognitive engagement depends not only on individual ability but also on the technological conditions under which knowledge work is performed. When AI assumes the cognitive operations that metacognitive monitoring would ordinarily track, the raw material for monitoring disappears; when AI is perceived as reliable, the perceived necessity of monitoring declines (de Visser et al., 2016). This dual erosion mechanism provides a more specific account of how AI use can reduce cognitive engagement, going beyond existing discussions of metacognitive laziness in learning contexts (Fan et al., 2025).

It is also noteworthy that cognitive offloading only partially mediated the effect of AI assistance on metacognitive disengagement and the effect of cognitive offloading on innovation quality. This partial mediation indicates that other mechanisms are likely involved. For example, when creators delegate primary cognitive responsibility to AI, their self-efficacy may shift from a sense of having solved the problem themselves to a sense of merely supervising the AI that solved it, and their sense of accountability for the output may weaken. These possibilities are consistent with the automation-augmentation paradox identified in management research (Raisch and Krakowski, 2021), in which the same AI system can either substitute for human cognition or augment it depending on how it is integrated into work processes.

### 7.2 Theoretical contributions

This research contributes to three bodies of literature: cognitive offloading theory, metacognition and AI literacy research.

First, we extend cognitive offloading theory from routine cognitive tasks to the metacognitive level of knowledge innovation. Prior research has primarily examined how individuals offload memory, navigation, or calculation to external tools and whether such offloading affects task performance (Risko and Gilbert, 2016). The present study reveals a different kind of offloading: generative AI enables creators to offload reasoning, synthesis, and evaluation, and the key cost of this offloading is located not at the task level but at the metacognitive level. By specifying how offloading reduces both the raw material for and the perceived necessity of metacognitive monitoring, we shift the focus of cognitive offloading theory from the behavioral delegation of tasks to the cognitive withdrawal from self-regulation. This reconceptualization offers a new entry point for understanding how technology-mediated knowledge work can gradually weaken higher-order cognition (Risko and Gilbert, 2016).

Second, we bring metacognition theory into the context of human–AI collaboration and identify the technological conditions under which metacognitive engagement recedes. Existing metacognition research has largely focused on individual learning and problem solving. By operationalizing metacognitive disengagement in human–AI collaborative knowledge innovation, we show that metacognitive decline can arise not from individual deficits but from the structure of the collaborative environment. Moreover, the moderated mediation pattern observed in Experiment 4 indicates that the formation of metacognitive disengagement is fairly general under high AI assistance, but its consequences depend on task characteristics and individual capabilities. This moves metacognition research beyond the question of whether metacognitive decline occurs to the question of when such decline translates into actual performance loss. The finding that task complexity strengthens the negative indirect effect is consistent with the principle that complex tasks demand more extensive cognitive processing (Campbell, 1988), while the buffering role of AI literacy suggests that metacognitive vigilance can be preserved through individual capability.

Third, this research contributes to the emerging literature on AI literacy by identifying a metacognitive protection function. Existing AI literacy frameworks emphasize technical competence, information evaluation, and ethical awareness (Long and Magerko, 2020; Ng et al., 2021). Recent work has further highlighted the metacognitive dimension of AI literacy, developing scales for collaborative AI metacognition (Sidra and Mason, 2025). Our findings suggest that the protective value of AI literacy is not primarily about using AI more skillfully, but about using AI more critically. High-AI-literacy individuals did not necessarily offload less; rather, they maintained greater critical distance from AI outputs and stronger metacognitive vigilance. This result implies that AI literacy education should move beyond tool operation and toward the cultivation of cognitive strategies that help individuals preserve their own thinking while collaborating with AI.

### 7.3 Practical implications

The findings have concrete implications for the design of AI systems, the organization of knowledge work, and the development of AI literacy.

First, AI systems intended to support knowledge innovation should incorporate metacognitive triggers rather than aiming for seamless substitution. For instance, before generating a solution, a system could ask users to articulate their initial assumptions; after generation, it could prompt users to mark which elements require verification or to compare at least one alternative; and at the end of a task, it could invite users to reflect on which judgments should remain under human control. These lightweight interventions re-embed planning, monitoring, and reflection into human–AI workflows, thereby reducing the likelihood that metacognitive disengagement will take hold. This approach aligns with research suggesting that external constraints can support rather than suppress creative cognition (Amabile, 1983) and with hybrid regulation frameworks that seek to balance AI support with learners’ self-regulation (Molenaar, 2022).

Second, AI use should be differentiated according to task complexity. Experiment 4 showed that metacognitive disengagement is especially damaging when tasks are complex. For knowledge work that requires cross-domain integration, multiple-constraint reasoning, or high uncertainty, organizations should be cautious about high-proportion AI generation. Structuring such work into phases, such as independent human ideation, AI-assisted expansion, and critical human integration, can help ensure that the key stages of problem framing, solution evaluation, and final selection remain under human metacognitive control. For simpler tasks, by contrast, a higher degree of AI assistance may be acceptable because the cost of reduced metacognitive engagement is lower.

Third, AI literacy education should shift from operational training toward critical and metacognitive training. Learners and knowledge workers should be trained to identify the assumptions and potential biases in AI outputs, to propose at least one alternative before accepting an AI-generated solution, and to reflect after task completion on which aspects of the work should remain under human judgment. Such training aligns with the view that critical evaluation is the core distinctive dimension of AI literacy (Ng et al., 2021; Long and Magerko, 2020) and can help individuals maintain metacognitive vigilance in AI-rich environments.

### 7.4 Limitations and future directions

Several limitations should be acknowledged. First, a critical limitation concerns the temporal scope of the present measure. This research focuses on task-level metacognitive disengagement, and the nine-item scale captures the immediate, task-specific reduction in planning, monitoring, and reflection within a single AI-assisted task rather than a habitual, cumulative decline. The experiments therefore demonstrate immediate reductions in metacognitive engagement but cannot determine whether such reductions persist or accumulate over time. Future research should employ longitudinal or repeated-exposure designs to examine this developmental trajectory.

Second, the experiments relied on online samples and short, simulated tasks. This design supports causal inference but limits ecological validity, as real innovation work is longer in duration, more socially embedded, and subject to different accountability structures. The findings may therefore not generalize to high-stakes, long-cycle projects, and effect sizes may differ. Future research could use field experiments or experience sampling methods to examine metacognitive disengagement in actual knowledge work settings (Dell’Acqua et al., 2026).

Third, the cognitive offloading manipulation in Experiments 3 and 4 was compound in nature. The high- and low-delegation conditions differed not only in the degree of cognitive delegation but also in AI response detail, independent effort requested, and responsibility allocation. The findings therefore reflect the combined influence of a high-delegation workflow, with cognitive offloading as its central feature. Future research could hold these features constant while varying only the degree of delegation to isolate the unique effect of cognitive offloading.

Fourth, knowledge innovation quality was assessed through expert ratings. Although interrater reliability was acceptable, expert judgment remains partly subjective. Future studies could incorporate more objective indicators, such as the originality of proposals, downstream implementation outcomes, or peer evaluations.

Fifth, cognitive offloading served only as a partial mediator. The present model focuses on cognitive offloading and metacognitive disengagement because they represent the most proximal mechanisms linking AI assistance to innovation quality. However, the residual direct effect indicates that other mechanisms are also involved. Future research could examine additional mediators such as trust in AI (Jiang et al., 2025), perceived responsibility (Yousefi et al., 2026), and creative psychological ownership (Ma et al., 2026), and test multiple-mediator models for a more complete account.

Sixth, although we conducted Harman’s single-factor tests in Experiments 2, 3, and 4, this approach is a relatively weak test of common method variance. Because all measures were self-reported, the mediation effects observed in Experiment 2 may be partly inflated by common method variance. Future research could employ CFA-based common latent factor models, multi-source ratings, or objective behavioral measures, such as platform analytics data, to further validate the proposed relationships.

Finally, the present study did not directly test interventions to reduce metacognitive disengagement. The buffering effect of AI literacy was measured as an individual difference rather than experimentally manipulated. Future research should design and evaluate interventions such as metacognitive prompting, structured human–AI workflow separation, or targeted AI literacy training. Given recent work on hybrid human–AI regulation (Molenaar, 2022) and the augmentation potential of AI (Jia et al., 2024), such interventions may help organizations capture the efficiency benefits of AI while preserving the cognitive engagement that sustains innovation quality.

## 8 Conclusion

This research reveals a hidden cognitive cost behind the efficiency gains of human–AI collaboration in knowledge innovation. Metacognitive disengagement reminds us that the most important risk of generative AI may not be that machines replace human thinking, but that humans gradually reduce the monitoring and reflection that keep their own thinking alive. Understanding how to maintain metacognitive engagement while benefiting from AI support is therefore a central challenge for the future of knowledge work.

## Data Availability

The raw data supporting the conclusions of this article will be made available by the authors, without undue reservation.
