# Supplementary material for: Metacognitive disengagement and knowledge innovation quality in human–AI collaboration: the mediating role of cognitive offloading and the moderating roles of task complexity and AI literacy
Source: Front Psychol. 2026 Oct 28;17:1975833. doi: 10.3389/fpsyg.2026.1975833 (PMC13751439; doi:10.3389/fpsyg.2026.1975833)
Supplement: Supplementary file 1 [file Supplementary_file_1.docx]

# Appendix A: Supplementary Measurement and Analysis Information

## Part 1: Measurement for this study

All scales in this study were rated on a 7-point scale (1 = strongly disagree, 7 = strongly agree), with higher scores indicating higher levels of the measured construct.

**Task-level Metacognitive Disengagement Scale (Used in Experiments 1, 2, 3, and 4)**

**Planning Reduction (3 items)**

In this task, I rarely spent time planning the steps to solve the problem.

Before starting the task, I did not develop a clear plan.

I did not actively consider different strategies I could use.

**Monitoring Weakening (3 items)**

In this task, I did not continuously check whether my thinking was correct.

During the task, I did not promptly detect misunderstandings in my reasoning.

I did not regularly evaluate my progress on the task.

**Reflection Absence (3 items)**

After completing the task, I did not review and summarize my thinking process.

I did not reflect on whether the strategies I used were effective.

I did not think about how to improve my problem-solving approach next time.

**Scoring:** Item scores were averaged, with higher scores indicating greater metacognitive disengagement.

**Source:** Developed for this study. Inspired by the measurement approach to metacognitive laziness in learning contexts (Fan et al., 2025) and adapted to the knowledge innovation context. The three dimensions (planning reduction, monitoring weakening, reflection absence) are grounded in classic metacognition research (Flavell, 1979; Schraw & Moshman, 1995; Winne & Hadwin, 1998).

**AI Assistance Manipulation Check (Used in Experiments 1 and 2)**

The AI assistant provided me with substantial help.

The AI assistant undertook a large part of my thinking work.

The AI assistant provided substantive suggestions.

**Scoring:** The three item scores were averaged, with higher scores indicating greater perceived AI assistance.

**Purpose:** Used to verify the effectiveness of the AI assistance manipulation.

**Cognitive Offloading Scale (Used in Experiment 2)**

In this task, I delegated a large part of my thinking to the AI.

In this task, I let the AI complete content that required mental effort.

In this task, I reduced my own effort in processing complex information.

In this task, I used the AI to reduce my cognitive load.

**Scoring:** Item scores were averaged, with higher scores indicating greater cognitive offloading.

**Source:** Adapted based on the conceptualization of cognitive offloading by Risko and Gilbert (2016).

**Cognitive Offloading Manipulation Check (Used in Experiments 3 and 4)**

In this task, I delegated my thinking work to the AI.

In this task, I reduced my own thinking effort.

In this task, I let the AI complete the core content.

**Scoring:** Item scores were averaged, with higher scores indicating greater cognitive offloading.

**Purpose:** Used to verify the effectiveness of the cognitive offloading manipulation.

**Task Complexity Manipulation Check (Used in Experiment 4)**

This task was very complex.

Completing this task required a great deal of cognitive effort.

This task involved multiple factors that needed to be weighed.

**Scoring:** Item scores were averaged, with higher scores indicating greater perceived task complexity.

**Purpose:** Used to verify the effectiveness of the task complexity manipulation.

**Individual AI Literacy Scale (Used in Experiment 4)**

**AI Knowledge (2 items)**

I understand the basic working principles and boundaries of AI systems.

I know in which aspects AI systems may perform poorly.

**AI Use Skills (2 items)**

I can effectively interact with AI systems to complete tasks.

I can adjust the way I ask questions to AI according to my needs.

**Critical Evaluation of AI (2 items)**

I can identify potential biases and limitations in AI outputs.

I critically evaluate the information or suggestions provided by AI.

**Scoring:** Item scores were averaged, with higher scores indicating greater AI literacy.

**Source:** Developed with reference to the AI literacy framework of Long and Magerko (2020).

## Part 2: Variable Coding for Analyses

| Variable | Coding |
| --- | --- |
| Cognitive offloading condition | 1 = high delegation, 0 = low delegation |
| Task complexity | 1 = high complexity, 0 = low complexity |
| Metacognitive disengagement | Continuous (mean of 9 items) |
| Knowledge innovation quality | Continuous (mean of 3 expert-rated dimensions) |
| AI literacy | Continuous (mean of 6 items, mean-centered in regression models) |
| Gender | 1 = male, 2 = female |
| Age | Continuous (years) |
| Education | 1 = high school or below, 2 = undergraduate, 3 = postgraduate |
| AI familiarity | Continuous (7-point scale) |

## Part 3: PROCESS syntax for Experiment 2, 3 & 4

Experiment 2 (Model 4: AI assistance → cognitive offloading → metacognitive disengagement)

PROCESS VARIABLES = Y X M

/Y = Meta_disengagement

/X = AI_condition

/M = Cognitive_offloading

/MODEL = 4

/BOOT = 5000

/CENTER = 1.

Experiment 3 (Model 4: cognitive offloading → metacognitive disengagement → knowledge innovation quality)

PROCESS VARIABLES = Y X M

/Y = KI_quality

/X = CO_condition

/M = Meta_disengagement

/MODEL = 4

/BOOT = 5000

/CENTER = 1.

Experiment 4 (Model 14: moderated mediation with task complexity and AI literacy as moderators)

PROCESS VARIABLES = Y X M W

/Y = KI_quality

/X = CO_condition

/M = Meta_disengagement

/W = Task_complexity

/MODEL = 14

/BOOT = 5000

/CENTER = 1.

PROCESS VARIABLES = Y X M W

/Y = KI_quality

/X = CO_condition

/M = Meta_disengagement

/W = AI_literacy

/MODEL = 14

/BOOT = 5000

/CENTER = 1.

# Appendix B: Exploratory Factor Analysis of the Task-Level Metacognitive Disengagement Scale

| Item | Factor 1 | Factor 2 | Factor 3 |
| --- | --- | --- | --- |
| Planning reduction |  |  |  |
| 1. In this task, I rarely spent time planning the steps to solve the problem. | .82 | .11 | .08 |
| 2. Before starting the task, I did not develop a clear plan. | .79 | .09 | .12 |
| 3. I did not actively consider different strategies I could use. | .75 | .14 | .10 |
| Monitoring weakening |  |  |  |
| 4. In this task, I did not continuously check whether my thinking was correct. | .12 | .81 | .09 |
| 5. During the task, I did not promptly detect misunderstandings in my reasoning. | .08 | .78 | .13 |
| 6. I did not regularly evaluate my progress on the task. | .15 | .74 | .07 |
| Reflection absence |  |  |  |
| 7. After completing the task, I did not review and summarize my thinking process. | .10 | .08 | .83 |
| 8. I did not reflect on whether the strategies I used were effective. | .13 | .11 | .79 |
| 9. I did not think about how to improve my problem-solving approach next time. | .07 | .09 | .76 |
| Eigenvalue | 4.21 | 1.87 | 1.34 |
| % of variance explained | 46.78 | 20.78 | 14.89 |
| Cumulative % of variance explained | 46.78 | 67.56 | 82.45 |

Note. N = 194 (Study 1, Phase 2). Extraction method: principal axis factoring. Rotation method: direct oblimin. Factor loadings above .40 are shown in bold. KMO = .91; Bartlett’s test of sphericity: χ²(36) = 1,245.67, p < .001. The three-factor solution accounted for 82.45% of the total variance. Factor intercorrelations: F1–F2 = .48, F1–F3 = .45, F2–F3 = .51.

# Appendix C: Measurement Invariance Across Experiments

To examine whether the scale functioned equivalently across the four experiments, we conducted measurement invariance tests using the combined sample (N = 728). Table S1 presents the fit indices for configural, metric, and scalar invariance models. Configural invariance was first tested by fitting the three-factor model separately across the four experiments, which showed acceptable fit (CFI = .94, RMSEA = .07). Metric invariance was then tested by constraining factor loadings to be equal across experiments; the change in fit was negligible (ΔCFI = .008, ΔRMSEA = .004). Scalar invariance was tested by additionally constraining intercepts to be equal; again, the change in fit was negligible (ΔCFI = .009, ΔRMSEA = .005). These results support configural, metric, and scalar invariance, indicating that the scale can be compared meaningfully across the four experiments.

Table S1 Measurement Invariance Tests Across Four Experiments

| Model | χ² | df | CFI | RMSEA | ΔCFI | ΔRMSEA |
| --- | --- | --- | --- | --- | --- | --- |
| Configural | 312.45 | 96 | .94 | .07 | — | — |
| Metric | 341.28 | 108 | .93 | .07 | .008 | .004 |
| Scalar | 376.52 | 120 | .92 | .07 | .009 | .005 |

Note. N = 728. All models showed acceptable fit. ΔCFI < .01 and ΔRMSEA < .015 indicate support for invariance.
